# The American Dental Society of Europe

**Published:** 1890-12

**Authors:** 


### THE AMERICAN DENTAL SOCIETY OF EUROPE.—SEVENTEENTH ANNUAL MEETING, PARIS, AUGUST 6 AND 7, 1889.

(Continued from page 645.)

*First Day.—Afternoon Session.—(Continued.)*

Dr. L. C. Bryan, Basel, presented a number of novelties in dental instruments of his own invention and construction, among which was an *air-pump* for driving a pneumatic mallet which was adjustable to any dental engine. The driving wheel of the pump was provided with a rubber tire, similar to a bicycle wheel, and the entire pump was suspended by a set screw which clasped the standard of the engine when not in use, just above the driving wheel of the engine. By letting the driving wheel of the pump down upon the engine wheel, the friction puts the pump in motion. The force of the blow was regulated by any one or two of these contrivances,—viz., lengthening or shortening the stroke of the piston by a set screw on the driving wheel attached to the bar of the piston, lengthening the cylinder which telescoped, or allowing the escape of air by a valve on the head of the cylinder.

Any of the pneumatic mallets may be run by the pump, which has nozzle attachments for two rubber tubes, but Dr. Bryan prefers the Rauhe angle mallet, to which he has adapted fifteen extra points to meet a larger variety of cases, the set of ten points sold with the mallet being inadequate. The blows are delivered with great rapidity by ordinary pedal motion of the dental engine, and can be regulated as above, from the finest and lightest blow desired to a blow which will drive a chisel point for breaking off refractory enamel corners or drive wedges between teeth. The fatigue of working the mallet in long operations is said to be greatly less than in treading the usual rubber ball.

A set of flexible nerve-canal reamers was the result of various improvements in the Petit reamers, the set of twelve having been made by the S. S. White Dental Manufacturing Company, after patterns and drawings furnished by Dr. Bryan. The shank of the *triangular* point is rounded and smaller than its largest cutting diameter. This prevents injury and disagreeable jarring of the enamel walls when drilling out root-canals where the whole or part of the crown remains.

Two other devices presented were a novel *rubber-dam holder*, with two clamps for holding the dam in place and supporting a napkin over the chin to protect it from the wet rubber, or drawing the dam taut in four directions to better expose the teeth operated on, and a *screw device* for adjusting to any rubber-dam clamp to prevent the clamp from impinging on the gum, the screw being out of the way of the operator in any operation, and being adjustable to a tooth of any length. With this device, and generally without it, Dr. Bryan finds the Southwick clamp, S. S. White Manufacturing Company, adjustable to any molar of either jaw, and has for two years used no other molar clamp.

Dr. Bryan called the attention of the members to some sample bottles of Hood & Reynolds's *gold cylinders*, prepared from corrugated foil of exceeding softness, and capable of great cohesion when annealed, superior for the rotation method and general use. He states that the German foil, although it is labelled No. 4 foil, is really No. 1 foil or No. 4 foil beaten out to four times the size of a usual sheet of No. 4. They have preserved their secret by labelling it No. 4 foil, leaving the public to infer that its softness is the result of some special process. Hood & Reynolds have made this foil for years, but the visit of Dr. Herbst to America advertised the German foil, and it is not generally known that the thin, soft, corrugated foil cylinders, recommended by Herbst, were first made in America, and are superior to the German cylinders in cohesion when annealed and of the same softness when unannealed. It is also tougher, and the mallet does not chop it up when annealed.

*Dr. Elliott.*—Dr. Mitchell has just reminded me that the American Dental Association meets to-day at Saratoga Springs, and I propose that a cable telegram of greeting be forwarded from this Society. This was adopted and the following sent:

"The American Dental Society of Europe, in session, sends fraternal greeting. DR. PATTON, *Secretary.*"

Dr. Miller then read a paper on pathological conditions in ivory with demonstrations on the black-board.

DISCUSSION ON DR. MILLER'S FIRST PAPER.

*Dr. Mitchell.*—I should like to ask Dr. Miller, what are the microscopical differences between nodular calcification of the pulp in elephant's teeth and that in human teeth, or if there is any similarity between those nodular pulp deposits and those found in human teeth?

*Dr. Miller.*—As far as my observation goes, the formation in the pulp of the tusks of elephants are identical with those occurring in the human teeth.

*Dr. Bryan.*—May not these pulp stones be considered as necessary nuclei for the rapid filling of the canal? This specimen here seems to have something on the surface of the tooth which stimulated the formation of them. We can see that the formation is not followed, and they close up the space entirely from this part without reference to the filling on the side.

*Dr. Miller.*—If you examine it under the microscope, you will find it nearly normal, the structure in which these pieces is embedded is quite different from the pieces themselves, and we find almost the structure of normal dentine. This is an abnormal case.

*Dr. Elliott.*—Now, gentlemen, our time has elapsed. Is there any gentleman present willing to offer himself as a sacrifice to Dr. Bonwill? Some one having a large cavity preferred. Before we adjourn, I would like to mention that we still have two papers from Dr. Miller and Dr. Patton. Dr. Bryan has something to exhibit. Dr. Chamberlain, of Rome, is not present; neither is my brother.

*Dr. Bryan.*—Your brother wished me to make his excuses.

*Dr. Elliott.*—Then we have a paper by Dr. Haskell, which has been, unfortunately, left behind, but Dr. Mitchell knows the salient points of it and will give us them to-morrow.

The meeting here adjourned until Wednesday morning.

*Wednesday, August 7, 1889.*

### COMBINATION FILLING AND ECLECTIC PRACTICE.

BY W. R. PATTON, D.D.S., COLOGNE, GERMANY.

[Dr. Patton's paper having been printed in the *Dominion Dental Journal*, an abstract is furnished in order that the discussion which follows may be understood.—Ed.]

The writer states that his experience during the past ten years has been that combination fillings are the most permanent,—gold and amalgam, gold and cement, amalgam and cement, tin and amalgam. He makes no mention of tin and gold, for the reason that he regards tin and amalgam as giving the same results with more ease in manipulation.

Oxychloride and oxyphosphate fillings would be the best form of material were it not for the liability of solution; but this forces the necessity for combination. In deep cavities, he fills in ninety-five cases with cement, and caps with gold or amalgam or both combined. The combination of gold and amalgam is, in his opinion, one of the best filling-materials. The dentine is more effectually preserved by it. His principle is to fill with gold wherever the stopping is visible, and the rest of the same cavity with amalgam. A prominent cavity on a central incisor would be filled on the labial surface with gold, and on the palatine with amalgam. The same treatment is recommended for other teeth. The writer then follows in review the "new departure" theories, and says, "Have plastic fillings had the same trial in all cases, in the same manner as gold?" In many places he regards plastic work as superior to gold, and follows with numerous instances, concluding as follows: "If we aim to relieve suffering and prolong the use of organs necessary to a healthy being, then searching for cause and observing effect, we must break the bonds of established usage and teaching, and, treading our way over new fields of extended and accurate observation, we may arrive at a goal where we will find that our efforts in plastic models may later be petrified into the permanent marble of success."

### DISCUSSION ON DR. PATTON'S PAPER.

*Dr. Mitchell.*—Mr. President and gentlemen, in these days of describing theories, I think it is a very good thing to listen to such a paper and to know that it is the outcome of practical experience. I think we accept preferably to-day a line of practice, follow it out, and our results, according to our capacities, are more or less satisfactory.

When we have been practising successfully for some little time a theory is advanced, breaking down all our pet hopes and schemes. Practice rather overrides theory, and through our patients we often find that certain proceedings are good ones or the reverse. Eclecticism is undoubtedly what we are steering for, and the man who steers close to it will not be entirely a gold or a cement filler, but will take what is best fitted for the case. One of our advanced teachers on the other side has made the remark, in a great many instances, that he has never used amalgam. Well, I do not think that he has been working to the best interest of himself or his patients.

More attention is being paid to therapeutics. Dr. Harlan has made a series of very elaborate experiments, and, according to our conception, some of the most highly prized. If we follow out his theories, we shall probably utilize more intelligently than we have done the application of remedies at our command. I think, in regard to all our lines of procedure, that the most practical man is a safely conservative one. He diagnosticates his case carefully, and upon that results mainly his success. The man who may know the merits and demerits of the different filling-materials, and will utilize them under different conditions, is the one fitted to practise. A case is to be followed out upon its merits, and by so doing, he will secure the best results to himself and to his patients.

*Dr. Elliott.*—Dr. Bonwill is here, and we will be glad to hear from him, however briefly, as he is very much interested in this particular line.

*Dr. Bonwill.*—I had hoped to have been permitted to listen, for, in my experience, I have done so much talking and working before societies that I have grown somewhat tired; but the subject you have before you is one which interests me very much, although I am looked upon as an extremist in every respect. It is well to hear what can be said upon an extreme side. I have learned that a good many things have been done, *not* wisely and *not* too well.

I have spent many years in trying to make men believe not that there is nothing else but gold, but that fillings in gold should be inserted, and that this can be done in a very short time. When I began, four hours could be spent on a single filling of a book of foil. I have seen many articles from gentlemen in this country, and often upon my own side, where they seem not to have any conception of machinery. My object was to reduce time, and save excessive nervous and muscular strain of the operator, and save the patient from the effects of the operation. In that respect, my work has accomplished all that I anticipated. When you come to consider the vast number of cases of large cavities now to what there were thirty years ago, and what I found in Philadelphia seventeen years ago, the difference is immense. Notwithstanding all these years spent with machinery for filling, I am filling more teeth with amalgam at the present time than I have ever done in my life.

Men forget that there is some one else besides themselves. We cannot afford to torture people with these large operations. I perceive very plainly that we must fill a large number of teeth with amalgam, and where the packing of amalgam is understood there will be a better system of practice, a larger number of teeth saved, and a larger amount of contour work done.

We cannot hurry through operations like you do in this country; it is not money alone that is to be considered; not the number of patients you must have in a day to make the most out of. Eclecticism in practice is to be recommended. My practice is now and then a gold filling in front teeth; I very seldom use gold unless I have a large operation to build up. Sometimes I fit a crown to a tooth, but there are thousands of crowns grafted on teeth which are of no value. I consider it the worst possible practice. A crown may come in now and then when a tooth cannot be stopped with gutta-percha. Bridge-work is valuable, but not always available. Bridge-work is advertised over here to gain reputation. I have found gutta-percha one of the best means of preserving teeth, especially children's teeth. Amalgam is not so satisfactory for children's teeth; it is difficult to keep the cavity dry; but gutta-percha can be packed in, and it is satisfactory. A point of eclecticism which I regard as most important is to get ourselves ingratiated with the children so that we can have them better subjects in the future. I very seldom use a gold filling in a child's tooth. I would not do it. I would not fill the teeth of children at the age of six or seven years with gold. I am getting more and more in love with amalgam. I save hundreds of teeth, and I think I do as much good in that way as in any other. Tin and gold is something I have never attempted. If I have to use tin, I use it exclusively; some of the early operations which I put in for my brother are in to-day, perfect. I would not mix gold with them, with the expectation that there would be less possibility of decay. If I use gold I use it exclusively. What effect would there be in mixing tin with gold? If tin and gold would make a harder filling, one which would preserve the contour of the tooth, I would say use it, and not tin; but I have seen these operations; they wear away if made ever so perfectly. Tin will preserve it just as well, or Abbey's adhesive No. 20 foil.

*Dr. Miller.*—I cannot allow this subject to pass by without saying something about it. I wish to take issue with Dr. Bonwill on the point of tin and gold. I think those who have used tin and gold for a number of years, and who have seen what can be accomplished by the average operator, are convinced that Dr. Bonwill has not given tin and gold a fair trial. Tin and gold has been used now for some forty or fifty years; it appears to have been first employed in England; however, Dr. Abbott has the merit of introducing tin and gold to the profession. Every one knows what Dr. Abbott has accomplished with it, and he advocated the use of it in the beginning of the history of this Society. Dr. Sachs, Dr. Jenkins, and others have used it extensively.

We find a great many places where it can be inserted with the greatest advantage. There are places where it is preferable to copper amalgam, although I am myself a great advocate of this latter material. As to the idea that tin is as hard as tin and gold, that is not the case, a tin-and-gold filling will become much harder than tin alone. I do not care what Dr. Bonwill has been able to accomplish himself in the use of gold. We want to know what the average dentist can accomplish with the various materials. This material has been introduced in the last few years in Germany, where the skill in manipulation is not so great as that arrived at in America. I think our friend, Dr. Sachs, and also Dr. Jenkins, will have something to say on the subject of tin-gold. What I wish to emphasize in particular is, that a great many dentists cannot arrive at the skill which is necessary for gold fillings, particularly in large cavities. I fill all large cavities extending under the margin of the gum with tin and gold. Of course, we know that we cannot always prepare the cavity properly for gold. No one, not even Dr. Bonwill, in cases of very extensive caries in soft teeth, can with certainty insert the gold so as to obtain a perfect filling, or one which does not soon show leakage, particularly at the neck. A better result is obtained in such cases by a tin-gold filling, or by a tin-gold filling with a gold cap.

*Dr. Jenkins.*—"Never measure another man's corn by your own bushel;" if one is considering eclectic practice, one must consider the person who is practising. I suppose it makes very little difference to us with what Dr. Bonwill would fill a cavity, for whatever material he selected, he would manipulate it in such a way as to produce a good result; but another man, in his position, would merely select such a material, but by working in his own way he would obtain a better result than he would in Dr. Bonwill's way. I would say that, in a German practice, especially a practice which extends over a wide territory, where frequently patients can be seen only once in several years, a man must use remarkable judgment as to what material he should employ in each case. A man has to make a great many operations which are not absolutely ideal, or would not be ideal if he could have that patient under inspection every two or three months. For cases where the patient can seldom be seen, where the teeth are soft, where there are no well-established habits of cleanliness, tin and gold seem to me absolutely indispensable. As far as I know, it is the only material used in filling the teeth which may with age improve. If a tin-and-gold filling is well made, it is a better and a stronger filling in one year, or in ten or twenty, than it was three months after it was inserted.

Dr. Miller has carefully explained in previous communications to the Society the scientific reasons for this curious molecular change. Sometimes we have to make a tin-and-gold filling in a half-erupted molar, where it is impossible to apply the rubber dam. I know of no material which can be used under these circumstances except soft gold, to which he has referred, and which can, in any case, be as reliable as tin and gold. I remember such gold fillings made twenty-four years ago, saturated through and through with moisture doing their work, but they were made in good material. Soft gold fillings made by the most skilful operator will not last as those prepared from tin and gold. The rapidity is a very important point. Contouring can only be done where the walls are strong, and where the operator is accustomed to working it in pellets or strips in the form of rolls from the bottom of the cavity to the top.

*Dr. Daboll.*—Will Dr. Miller be kind enough to again explain his mode?

*Dr. W. D. Miller.*—We have not much time, so I will endeavor to explain this matter as briefly as possible. Place a sheet of gold-foil in a glass vessel, and cover it with a one-per-cent. solution of lactic acid. Place upon the tin-foil a sheet of gold-foil, and upon this a small glass plate to keep it in position. In the course of about twenty-four hours we will find that the surface of the gold in contact with the tin has a color very much resembling that of the tin. In fact, sometimes it is scarcely possible to say which is gold and which is tin. A day or two later the color of the gold will be changed to brown, and in some cases to nearly black. If the experiment is made under pressure, the two sheets will adhere, stick firmly together, so that they cannot be readily separated. I have examined into the process very carefully, and have come to the conclusion that this phenomenon is brought about by an electrolytic solution of the tin and a redeposition upon the surface of the gold. It is not the tin which changes color, but the gold, the tin being gradually dissolved and deposited upon the gold, by which the two substances become thoroughly cemented together.

*Dr. Elliott.*—The next meeting, gentlemen, will take place in Heidelberg, on the first Monday in August, 1891.

The election for officers resulted as follows: President, Dr. Patton, Cologne; Vice-President, Dr. Davenport, Paris; Secretary, Dr. Bryan, Basel; Treasurer, Dr. Adams, Frankfort.

Adjourned.

To fitly close the proceedings of this meeting a dinner had been arranged by the Executive Committee on the Eiffel Tower, which proved very enjoyable. From there the members separated to all points of the compass, with hopes of meeting again at Berlin in 1890, at the International Medical Congress, or at Heidelberg in 1891.

W. R. Patton,
*Secretary.*

